# Duration analysis using matching pursuit algorithm reveals longer bouts of gamma rhythm

**DOI:** 10.1152/jn.00154.2017

**Published:** 2017-11-08

**Authors:** Subhash Chandran KS, Chandra Sekhar Seelamantula, Supratim Ray

**Affiliations:** ^1^Department of Electrical Engineering, Indian Institute of Science, Bangalore, India; ^2^Centre for Neuroscience, Indian Institute of Science, Bangalore, India

**Keywords:** beta, burst, gamma, matching pursuit, primary visual cortex

## Abstract

The gamma rhythm (30–80 Hz), often associated with high-level cortical functions, is believed to provide a temporal reference frame for spiking activity, for which it should have a stable center frequency and linear phase for an extended duration. However, recent studies that have estimated the power and phase of gamma as a function of time suggest that gamma occurs in short bursts and lacks the temporal structure required to act as a reference frame. Here, we show that the bursty appearance of gamma arises from the variability in the spectral estimator used in these studies. To overcome this problem, we use another duration estimator based on a matching pursuit algorithm that robustly estimates the duration of gamma in simulated data. Applying this algorithm to gamma oscillations recorded from implanted microelectrodes in the primary visual cortex of awake monkeys, we show that the median gamma duration is greater than 300 ms, which is three times longer than previously reported values.

**NEW & NOTEWORTHY** Gamma oscillations (30–80 Hz) have been hypothesized to provide a temporal reference frame for coordination of spiking activity, but recent studies have shown that gamma occurs in very short bursts. We show that existing techniques have severely underestimated the rhythm duration, use a technique based on the Matching Pursuit algorithm, which provides a robust estimate of the duration, and show that the median duration of gamma is greater than 300 ms, much longer than previous estimates.

## INTRODUCTION

Signals recorded from the brain often show rhythmic fluctuations at different frequencies, which are associated with different behavioral states (Buzsáki and Draguhn 2004; Buzsáki 2006). One such frequency band is gamma (center frequency between 30 and 80 Hz), which is modulated by high-level cognitive functions such as binding (Gray et al. 1989) and attention (Fries et al. 2001) and has been hypothesized to play a critical role in cortical processing (Fries 2009, 2015). An influential hypothesis, first posited for the theta rhythm (Buzsaki and Chrobak 1995; O’Keefe and Recce 1993) and later extended to gamma (Fries et al. 2007), is that rhythms provide a temporal reference for spiking activity in the brain, such that information can be coded in the position of the spike relative to the rhythm. For this mechanism, the frequency and phase progression of the rhythm should remain consistent for durations relevant for behavior.

Recent reports studying the consistency of power and phase of the gamma rhythm in the local field potential (LFP) recorded using microelectrodes have shown that in the primary visual cortex gamma occurs in bursts of very small duration (~120 ms) and has characteristics of filtered noise rather than a “clock” signal (Burns et al. 2011; Xing et al. 2012). A similar result was obtained for the beta rhythm in the striatum and premotor-motor cortex (Feingold et al. 2015), as well as for the beta and gamma activities in the prefrontal cortex of monkeys engaged in a working memory task (Lundqvist et al. 2016). Similar results were obtained from electrocorticogram (ECoG) recordings from primary visual cortex (Rols et al. 2001) from one monkey (mean duration of 88 ms), although the other monkey had longer bursts (mean duration of 254 ms). All of these studies estimated the burst duration by first computing the phase and/or power of the rhythm as a function of time, using a spectral estimator, finding “seeds” of bursts when the power exceeded a predetermined threshold, and finally measuring the time around the seed for which the phase progression remained consistent (Burns et al. 2011; Xing et al. 2012) or power remained high (Feingold et al. 2015; Lundqvist et al. 2016; Rols et al. 2001). This is a sound strategy if the spectral estimator is able to estimate the power/phase accurately, but unfortunately that is not the case. Direct spectral estimators are known to have high variance, with estimated power following an exponential distribution (Brillinger 1972; Jarvis and Mitra 2001) (see Fig. A1 of Srinath and Ray 2014), which masks the variability due to biological factors. The spectral estimation variability arises because we are trying to estimate the power spectral density, which is a continuous function, using finite data (see (Brillinger 1972; Jarvis and Mitra 2001) for details), and has no biological significance. However, because the estimated power follows an exponential distribution, any rhythm will always trivially appear bursty, with a few large values interspersed between many small values.

Here, we use a Matching Pursuit (MP)-based algorithm to estimate the gamma duration (Chaibi et al. 2013; Goelz et al. 2000; Jmail et al. 2011; Picot et al. 2011). In MP, a large number of oscillatory functions of different durations are available for representing the signal, and the algorithm picks the best function (and hence duration) based on the local properties of the signal. We show through simulations of gamma bursts of prespecified lengths that this MP-based algorithm does not suffer from the limitations of the other methods. Using this algorithm, we show that the median duration of gamma in signals recorded from the primary visual cortex of awake monkeys is greater than 300 ms, much longer than the value reported in most of the previous studies.

## MATERIALS AND METHODS

### 

#### Data acquisition.

Local field potential (LFP) signals were recorded from two adult female bonnet monkeys (*Macaca radiata*) of ages 13 and 17 yr. All procedures were approved by the Institutional Animal Ethics Committee (IAEC) of the Indian Institute of Science and the Committee for the Purpose of Control and Supervision of Experiments on Animals (CPCSEA). A 10 × 10 array of microelectrodes (96 active platinum electrodes, Utah array; Blackrock Microsystems) was surgically implanted under general anesthesia in the primary visual cortex (area V1) of the right cerebral hemisphere (~15 mm rostral from the occipital ridge and ~15 mm lateral from the midline) of each monkey. The receptive fields of the neurons recorded from the microelectrodes were centered in the lower left quadrant of the visual field at eccentricities between ~1.3 to ~4.5°.

The monkeys were required to fixate on a small spot (0.05° or 0.1°) on the center of a LED screen (BenQ XL2411, 1280 × 720 resolution, 100 Hz refresh rate; linearized for contrast) while large static gratings of radius 4.8° covering all the receptive fields were presented at five contrasts: 0, 25, 50, 75, or 100%, chosen pseudorandomly, at an orientation and spatial frequency that generated robust gamma rhythms in both monkeys (spatial frequency of 4 cycles per degree; orientation of 60° and 90° for *Monkey 1* and* Monkey 2*, respectively). In each trial, after acquiring fixation, a blank screen was shown for 2 s followed by a stimulus for 2 s, and the monkeys received a juice reward for maintaining fixation within 2° of the fixation spot for the entire duration. Stimulus at each contrast was repeated 102–104 times for *Monkey 1* and 77–96 times for *Monkey 2*. The LFP signals were acquired by filtering the raw signals between 0.3 Hz (Butterworth filter, 1st order, analog) and 500 Hz (Butterworth filter, 4th order, digital) and digitizing at 16-bit resolution with a sampling frequency of 2 kHz. We further applied a Chebyshev type I low-pass filter at 100 Hz and downsampled the LFP to 250 Hz to reduce the signal size using the “decimate” function in MATLAB. We selected 65 electrodes from *Monkey 1* and 39 electrodes from *Monkey 2* for which reliable estimates of receptive fields were obtained.

#### Generation of synthetic LFP.

We generated synthetic LFP signal by injecting Gabor atoms (sinusoid modulated by a Gaussian, representing gamma) of a fixed duration into the spontaneous LFP (signals recorded while presenting zero-contrast stimuli). We defined the length of the gamma burst to be four times the standard deviation of the Gabor atom (which covers ~95% of the energy of the Gabor atom). All simulations were based on data collected for the 100% contrast condition. We defined the stimulus period to be between 0.5 and 2 s after stimulus onset to avoid response transients. The position of each synthetic Gabor atom was chosen uniformly within the stimulus period with the condition that the entire burst should lie within the stimulus period (consequently, longer burst centers were confined toward the middle of the stimulus period). For each trial, the number of bursts injected was either one or obtained from a Poisson distribution with a mean equal to the ratio of the stimulus period and burst length, with the condition that the gamma bursts did not overlap (in case of overlap, one of the overlapping bursts was removed). For each burst, the center frequency was chosen from a uniform distribution between 40 and 60 Hz (because the gamma “bump” was mainly localized in this range for both monkeys). The amplitude of the burst was obtained from a normal distribution with a mean given by the difference in the magnitudes of average Fast Fourier transforms (FFT) of stimulus and spontaneous LFP at the chosen center frequency. The standard deviation of the amplitude was a free parameter, because the actual physiological variability is unknown [it is masked by the variability of the spectral estimator (Brillinger 1972; Jarvis and Mitra 2001)], and was set to 10% of the mean (i.e., a coefficient of variation of 0.1). All the synthetic LFP trials were then scaled with a constant such that the average synthetic LFP spectrum matched the real LFP spectrum in the gamma range (40–60 Hz) from a single electrode. Note that the actual LFP power spectrum has a broadband power increase before and after the gamma bump as well. This was not considered while simulating the synthetic signal, such that the simulated power spectra matched the real spectra only over 40–60 Hz but not at other frequencies (see Fig. 3*A*).

#### Burst detection using continuous Gabor transform.

The continuous Gabor transform (CGT)-based gamma burst detection algorithm (Burns et al. 2010 2011; Xing et al. 2012) has three steps. First, the spectrogram (Ackroyd 1971; Allen 1977; Allen and Rabiner 1977; Cohen 1995; Portnoff 1980) of the stimulus driven and spontaneous LFP were computed using the CGT:

$$G\left(t_{0},\omega_{0}\right)=\int f\left(s\right)\psi \left(s-t;t_{0};\omega_{0}\right)ds,$$

(1) $$\psi \left(t;t_{0},\omega_{0}\right)=\frac{1}{\sigma \sqrt{2\pi }}e^{\frac{-\left(t-t_{0}\right)}{2\sigma^{2}}}.e^{2\pi i\omega_{0}t}$$

where *G* is the CGT estimate at time *t_0_* and frequency *f_0_*, while Ψ is the complex Gabor filter with a standard deviation of σ.

Second, points in the time-frequency plane in the 40–60 Hz (gamma range) and after stimulus onset, which had power exceeding a particular threshold [Xing et al. (2012) used a threshold of three times the spontaneous power] were chosen as “seeds.” Finally, a phase consistency check was carried out around “seed” points such that the unwrapped and delinearized phase would lie within 45° of the phase at the seed point to obtain the duration of the gamma rhythm (see Xing et al. 2012 for details).

#### Burst detection algorithm used by Feingold et al.

In their technique of Feingold et al. (Feingold et al. 2015), the signal was first band-passed filtered in the frequency range of interest (13–30 Hz for their data) using a fourth-order Butterworth filter in both forward and reverse directions to ensure zero phase shift. The band-pass filtered signal was then squared and subsequently smoothed with a Hann window of suitable length (twice the time period of the lowest frequency content, i.e., 13 Hz in their case) to get the envelope. Occurrences of beta bursts were registered when the power exceeded three times the median power (seed points). The start and end of each burst were marked when the power went below 1.5 times the median power from that seed point. We have followed a similar procedure for gamma burst detection using the range 40–60 Hz instead of 13–30 Hz.

#### Burst detection using wavelet transform.

Burst detection using wavelet transform (WT) (Rols et al. 2001; Roux et al. 2007) is similar to the CGT-based method, except that the time-frequency power spectrum is computed using the WT and the burst length estimation is based only on power (and not phase) values. WT decomposes a signal into a series of orthogonal waveforms generated by scaling and shifting a single (“mother”) wavelet function in the time domain. The mother wavelet is well localized in time, has zero mean and unit energy, and satisfies some other conditions called “admissibility criteria” (Daubechies 1992; Mallat 2008; Vetterli and Herley 1992).

The continuous wavelet transform is defined as

(2) $$W_{f}\left(u,s\right)=\int f\left(t\right)\frac{1}{\sqrt{s}}\psi^{*}\left(\frac{t-u}{s}\right)dt$$

where *f*(*t*), ψ(*t*), *u*, and *s* are the signal, mother wavelet, translation factor and scaling factor, respectively, while $\frac{1}{\sqrt{s}}$ is the normalization constant and |*W_f_*(*u*,*c*)|^2^ represents the time-scale power distribution. Replacing scales with corresponding frequency values gives the time-frequency spectrum. In our implementation, we used the Morlet or Gabor wavelet, which uses a Gaussian window. The standard deviation of the Gaussian, which determines the length of the burst, is a free parameter that scales inversely with the center frequency. We have chosen a Q-factor (the ratio of center frequency to bandwidth, which remains a constant in WT) of ~9, which yields a standard deviation of ~28 ms at ~50 Hz (center of the gamma range). In this case, the estimated duration values were similar to the injected burst durations in synthetic data (Fig. 3). Note that increasing the Q-factor would trivially result in longer gamma durations.

#### Burst detection using Hilbert transform.

Burst detector using Hilbert transform (HT) (Lundqvist et al. 2016) is similar to the method used by Feingold and colleagues, except that the instantaneous power is calculated using HT by taking the square of the instantaneous amplitude. The instantaneous amplitude is the absolute value of the analytic signal, defined as

(3) $$A_{s}\left(t\right)=s\left(t\right)+jH\left\{s\left(t\right)\right\}$$

where *s*(*t*) is the band-pass filtered signal (same filter settings as used in the method by Feingold and colleagues) and *H* is the Hilbert transform.

#### Burst detection using matching pursuit.

Matching pursuit (Chandran KS et al. 2016; Durka et al. 2001; Mallat and Zhang 1993), originally proposed by Mallat and Zhang, is an iterative decomposition technique that approximates a time-domain signal to linear combination of waveforms called atoms. Here, a large and overcomplete dictionary of atoms is first created by shifting, scaling and modulating a mother atom:

(4) $$g_{\gamma }\left(t\right)=\frac{1}{\sqrt{s}}g\left(\frac{t-u}{s}\right)e^{j\xi t}$$

where *g*_γ_(*t*) is an element of the dictionary obtained by modulating the mother atom g(*t*) by γ = (*s*,*u*,ξ) that corresponds to a particular choice of scale (*s*), shift (*u*), and modulation (ξ) factors.

MP starts by setting the “residue” equal to the signal itself, computes the inner product between the residue and all the atoms in the dictionary, and selects the atom with the largest inner product. The signal is approximated as the chosen atom multiplied by the inner product between the atom and the residue. For the next iteration, the residue is the difference between the signal and the approximation, and the procedure is repeated. The residue after the *n*^th^ iteration is the difference between the signal and the decomposed approximation till the *n*^th^ iteration.

(5) $$f=\overset{m-1}{\underset{n=0}{\sum }}〈R^{n}f,g_{\gamma n}〉g_{\gamma n}+R^{m}f$$

$$g_{\gamma n}=arg\;max_{g_{\gamma i}\in D}\left|〈R^{n}f,g_{\gamma i}〉\right|$$

where *f(t)*, *R^n^f*, and *g_γ_n__* are the signal, *n*^th^ residue, and *n*^th^ selected atom, respectively.

We used MP algorithm with a stochastic dictionary (Durka et al. 2001), in which the scale, shift, and modulation parameters are sampled uniformly. The stochastic dictionary differs from the dyadic dictionary (which was originally used in Mallat and Zhang 1993), where the scale of the atom changes in powers of two. We did not use dyadic sampling, because the scale of the atom is related to its duration such that having only scales that are powers of two in the dictionary would obviously yield gamma durations that are also powers of two. With a stochastic dictionary, scales of the atoms (and hence durations) were sampled uniformly over a large range. The dyadic dictionary has *O*(*NlogN*) atoms, where *N* is the length of the signal (Mallat and Zhang 1993). However, the size of the stochastic dictionary does not depend on *N*, because the parametric space can be divided into arbitrary small divisions, which allows us to have different dictionary sizes for the same signal. We found that the default dictionary size of ~70,000 atoms for a signal of length 1,024 did not sample the scale, shift, and modulation parameter space densely enough, leading to an overestimation of gamma durations in Fig. 3. We therefore repeated the analysis with different dictionary sizes and found that accurate results were obtained for dictionary sizes of 300,000 atoms and above (~4 times the standard size). To ensure that our results were not biased by insufficient sampling of parameter space, all results shown here are computed for a dictionary size of 2,500,000 atoms (~35 times the standard size).

**Fig. 1.**
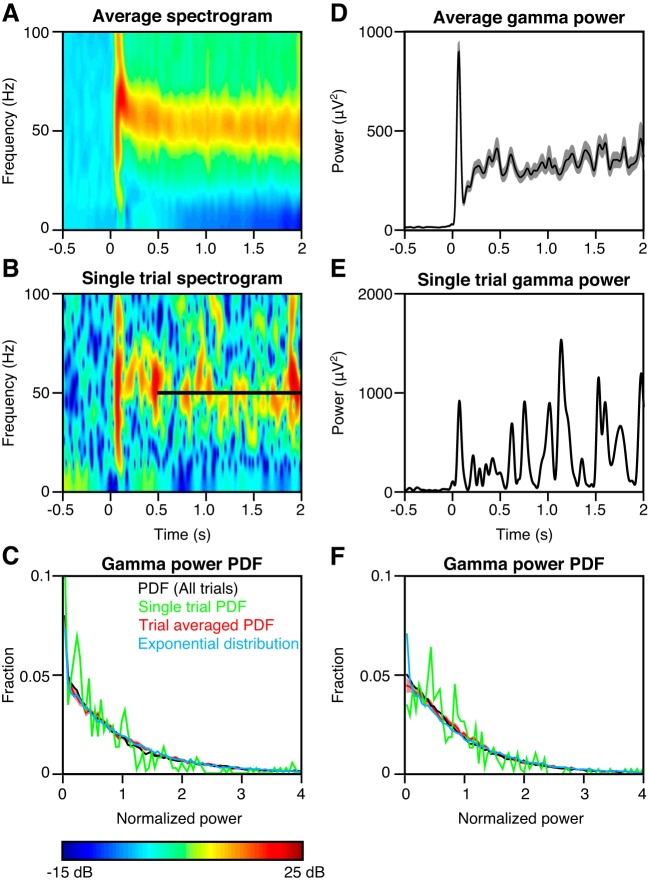
Variability in continuous Gabor transform (CGT) and gamma band power estimates. The trial averaged (*A*) and single trial change (*B*) in power [from spontaneous local field potential (LFP)] spectrogram of LFP recorded from a single electrode from *Monkey 1*, obtained using CGT. Black line at 50 Hz between 0.5 and 2 s indicates the points in the time-frequency plane for which power is chosen for computing the histograms. *C*: probability distribution function (PDF; obtained by computing a histogram and normalizing such that the total area is unity) of normalized power values for a single trial (green) and all trials (black). Power values were normalized to have a mean of unity. The red trace is obtained by normalizing the power values in each trial to have a mean of unity and therefore is the average of the single trials power spectral density (PSD; similar to the green trace) across trials. Lighter shade of red represents SE. Cyan trace shows the theoretical exponential distribution with a mean of unity. The trial averaged (*D*) and single trial gamma frequency band (*E*) (40–60 Hz) power, which was smoothed using a Hanning window, for the same data as shown in *A–C*. Gray shaded area in *D* shows SE of the mean. *F*: same as *C* for power values obtained from *D* and *E*.

**Fig. 2.**
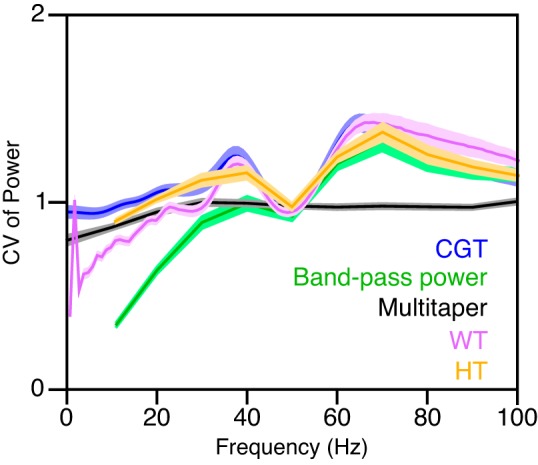
Spectral variability using different techniques. For the same data as in Fig. 1, coefficient of variation in power was calculated, separately for each trial, by first estimating the power at different time points, using the techniques described in the text, and then computing the standard deviation in power values between 0.5 and 1.5 s divided by the mean power in the same interval. Lighter shaded area shows SE of the mean, calculated across trials.

**Fig. 3.**
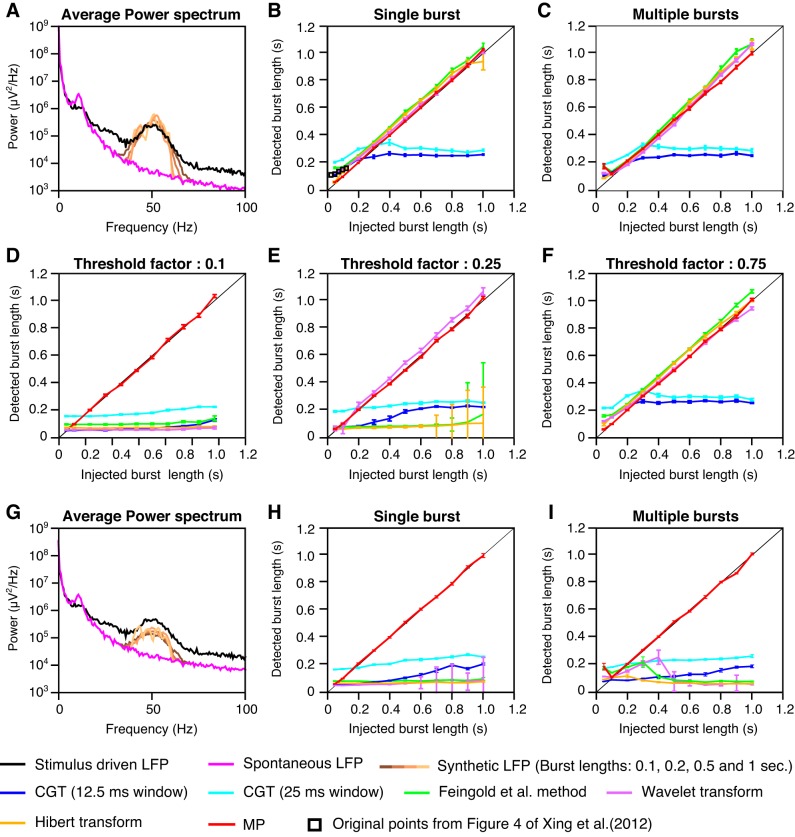
Comparison of gamma duration estimators. *A*: the trial averaged PSD of stimulus driven (black trace) and spontaneous (magenta trace) LFP signal recorded from a single electrode in *Monkey 1*, along with the PSDs of the stimulus-driven synthetic LFPs with a single gamma burst per trial (of durations 100, 200, 500, and 1,000 ms, shown in darker to lighter shades of brown). *B*: the injected vs. median estimated gamma durations using CGT-based algorithm [with a window length of 12.5 (blue) and 25 ms (cyan)], algorithm used by Feingold and colleagues (green), wavelet transform (WT; purple), Hilbert transform (HT; yellow), and matching pursuit (MP)-based algorithm (red) from synthetic LFP with a single burst. The threshold fraction (see text for details) was set to 0.5. Black squares indicate the points shown in Fig. 4 for Xing et al. 2012. *C*: same as *B*, but for multiple gamma bursts per trial. *D–F*: same as *B*, but when threshold fraction was set to (*D*) 0.1, (*E*) 0.25, (*F*) 0.75. *G*: average PSD of synthetic LFP when gamma power was scaled down to 25% of the original value. *H* and *I*: same as *B* and *C*, for synthetic LFP shown in *G*. Error bars represent the SE of median, computed using bootstrapping.

Because the total power in the LFP decreases with increasing frequency (Fig. 3*A*), typically low frequency atoms are chosen initially, such that a large number of iterations are needed to adequately capture the activity at high frequencies (including the gamma range). Therefore, to reduce the computational load, we first band-pass filtered the signal over 30–70 Hz using a 4th order Butterworth filter (applied in forward as well as reverse order using the command “filtfilt” in MATLAB) and performed MP decomposition. For a subset of data, we also performed the analysis on the unfiltered data while using a larger number of iterations; the results were similar. Atoms whose center frequency was in the gamma range, whose center position was in the stimulus period, and which had a coefficient larger than a fixed threshold were chosen as gamma bursts, and their corresponding scales were used to obtain the duration. Since the entire gamma atom is not constrained to lie within the stimulus duration (as opposed to previous methods), we occasionally obtained durations exceeding 2 s (5.7 and 6.8% of cases for the two monkeys). Visual inspection of such long gamma atoms along with the band-pass filtered LFP signal often showed transient fluctuations in the LFP power, even though the phases remained aligned near the center of the atom, along with occasional non-phase-aligned bursts near the tails of the atom. As discussed later, the actual gamma bursts could be more abrupt than the smooth Gaussian tapering assumed by the method, leading to an overestimation of the actual duration. Such atoms were therefore not considered for further analysis; including these atoms increased the gamma duration for both monkeys by ~30 ms. All data and codes required for generating Figs. 1–3 are available at: https://github.com/supratimray/GammaLengthProjectCodes.

Since the LFP power falls rapidly with increasing frequency, all the time-frequency power spectra are shown after baseline correction, as follows

(6) $$D\left(t,\omega \right)=10\times \left[log_{10}E\left(t,\omega \right)-log_{10}B\left(\omega \right)\right]$$

where *E*(*t*,ω) is the raw time-frequency spectrum while *B*(ω) is the mean baseline power obtained by averaging the power between −1.5 and 0 s, where 0 indicates stimulus onset. This has units of decibels (dB).

## RESULTS

**Fig. 4.**
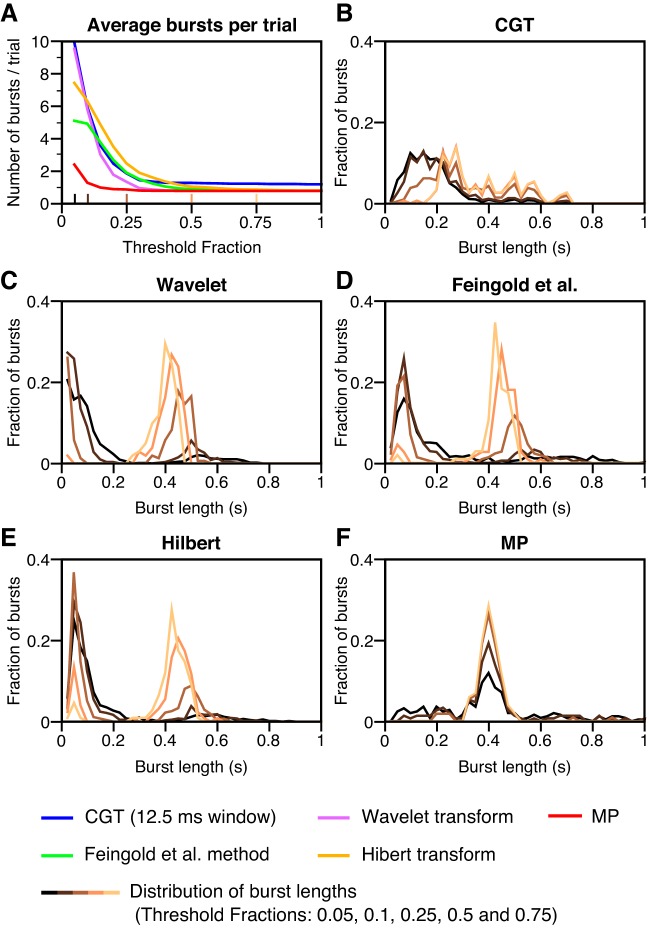
Analysis of number and duration of detected gamma bursts in synthetic LFP as a function of threshold fraction. For these simulations, a single gamma burst of 400 ms duration was injected per trial. *A*: average number of bursts detected per trial as a function of threshold fractions using CGT-based algorithm (blue), WT (purple), algorithm used by Feingold and colleagues (green), HT (yellow), and MP-based algorithm (red). *B–F*: probability distribution function of burst lengths detected for threshold fractions of 0.05, 0.1, 0.25, 0.5, and 0.75 (shown in darker to lighter shades of brown; also indicated on the abscissa in *A*, using (*B*) CGT-based algorithm, (*C*) WT, (*D*) algorithm of Feingold and colleagues, (*E*) HT, and (*F*) MP-based algorithm. For visual clarity, results are shown only up to 1 s.

**Fig. 5.**
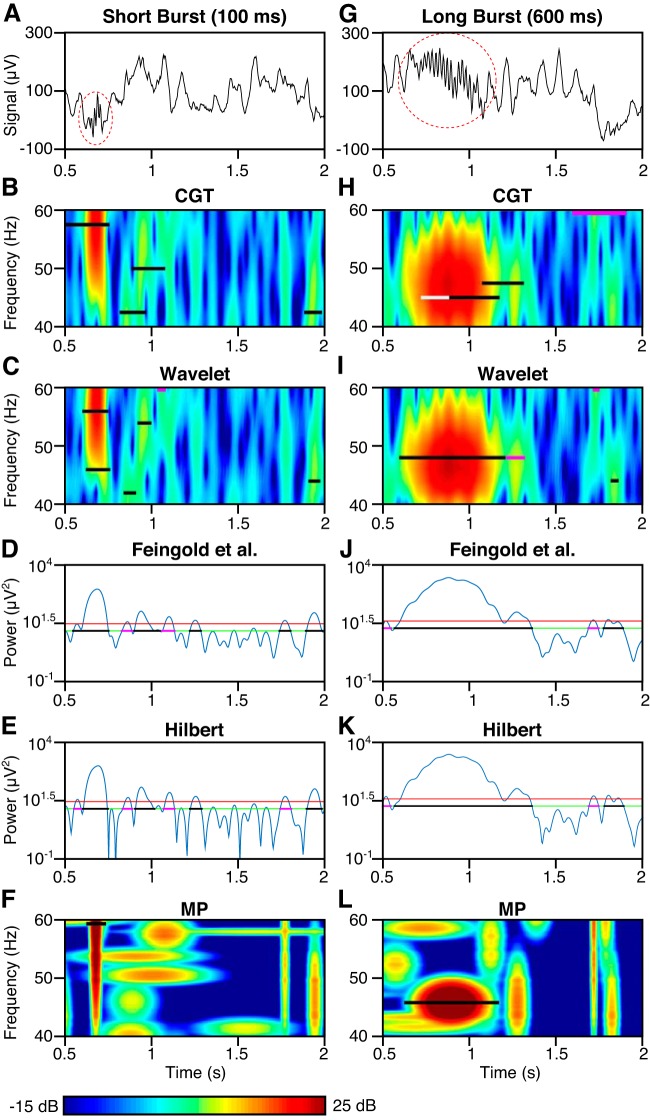
Gamma burst detection in a single trial of synthetic LFP. *A*: single trial synthetic LFP with short gamma burst (100 ms), marked with a dotted red ellipse. *B*: time-frequency change in power spectra of the synthetic signal shown in (*A*), using CGT, with a window length of 25 ms. Detected gamma bursts are marked with black, white, or magenta lines. Different colors are used only to distinguish between neighboring bursts. *C*: same as *B*, computed using WT. *D*: instantaneous power obtained by the algorithm used by Feingold and colleagues (blue trace), along with the threshold (red trace) above which the burst seeds were obtained, and 50% of the threshold (green trace) that were used to find the start and end of each burst; see materials and methods for details. *E*: same as *D*, using HT. *F*: same as *B*, using MP algorithm. *G–L*: same as *A–F*, for an injected burst of 600 ms duration.

**Fig. 6.**
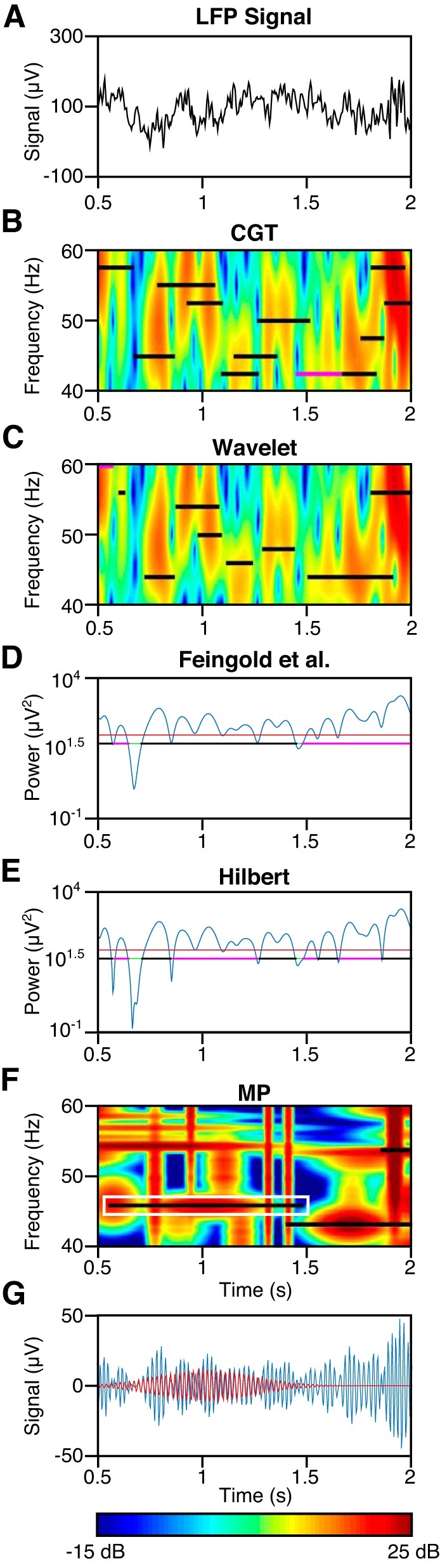
Gamma burst detection in a single trial of real LFP. *A–F*: same as *A–F* of Fig. 5, but for a single trial of real LFP signal. *G*: LFP signal shown in *A*, band-pass filtered between 40 and 60 Hz to highlight the gamma component (blue trace). The gamma atom highlighted with a white rectangle in *F* is shown in red.

**Fig. 7.**
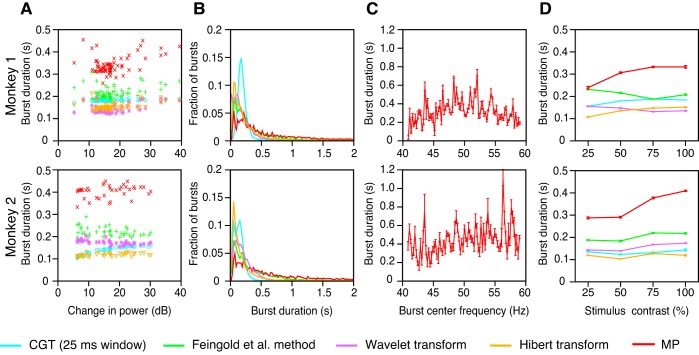
Duration of the gamma rhythm in real data. *A*: median gamma length per site as a function of the change in mean gamma power from spontaneous activity at that site, for 65 sites in *Monkey 1* (*top*) and 39 in *Monkey 2* (*bottom*), using CGT (cyan), Feingold’s method (green), WT (purple), HT (yellow), and MP (red). *B*: histogram of gamma durations from all sites for the two monkeys. *C*: median gamma durations computed separately at each center frequency using the MP algorithm. *D*: median gamma durations computed using different methods for different stimulus contrast levels. Error bars in *C* and *D* indicate SE of median, computed using bootstrapping.

The results are presented as follows. First, we compute the time-frequency power spectra or instantaneous power of real LFP signals using four methods that have been previously used to estimate burst duration, which are based on continuous Gabor transform (CGT; (Xing et al. 2012), a band-pass filtering technique used by Feingold and colleagues (Feingold et al 2015), wavelet transform (WT; Rols et al. 2001; Roux et al. 2007) and Hilbert transform (HT; Lundqvist et al. 2016), and show that the estimated gamma power follows an exponential distribution in all cases (Figs. 1 and 2). Next, we generate synthetic gamma bursts of known duration and test the performance of these four methods as well as an MP-based method (Figs. 3–5). Finally, we estimate the gamma burst duration using all five techniques in real LFP data (Figs. 6 and 7).

### 

#### Short gamma bursts in spectrograms are due to variability in the spectral estimator.

Figure 1*A* shows the average change in power from spontaneous activity (–1.5 to 0 s, where 0 indicates the stimulus onset) for 102 trials obtained from a single electrode from *Monkey 1*, computed using the CGT, following the same procedure as in (Xing et al. 2012). The average spectrum showed a robust gamma rhythm that was sustained for almost the entire duration of visual stimulation (2 s). In the single trial spectrogram, however, gamma seemed to appear in short bursts (Fig. 1*B*). However, this bursty-ness could have been due to the variability in the spectral estimator used to compute the spectrogram, which in many cases, is known to follow an exponential distribution (Brillinger 1972; Jarvis and Mitra 2001). To test this, we plotted a histogram of power values at 50 Hz (green trace in Fig. 1*C*) in the stimulus period (0.5 to 2 s; highlighted with a black line in Fig. 1*B*), which indeed followed an exponential distribution (cyan trace). This was observed when power values were pooled across trials (black trace in Fig. 1*C*) and even when power values for each trial were normalized to unity before averaging to remove variability in mean power across trials (red trace). The procedure used by Feingold and colleagues (Feingold et al. 2015) involves band-pass filtering the signal followed by smoothing to obtain a time series of power values (see materials and methods for details). However, band-pass filtering involves taking the inner product between the signal and a filter and is therefore similar to computing a spectral estimate. Consequently, the gamma band power series obtained using their technique (Fig. 1, *D* and* E*) was also found to be noisy and followed an exponential distribution (Fig. 1*F*). Similar results were obtained for other techniques (see Fig. 2; not shown in detail because these approaches are similar to the methods used in these two studies).

To study the variability in the spectral estimate across time at other frequencies for the same data as in Fig. 1, we computed the time-frequency power using CGT, WT as well as the multi-taper method (as used by (Lundqvist et al. 2016); we used a single taper with a length of 100 ms, which was shifted in steps equal to the sampling period), and estimated the coefficient of variation (CV; standard deviation divided by the mean) of power across time for each trial, separately at each frequency (Fig. 2). Even when the analysis period was restricted to 0.5–1.5 s to exclude transients related to both stimulus onset and offset, the mean CV of power was close to the theoretical value of 1 (CV of an exponentially distributed random variable) or higher at almost all frequencies (note that a CV greater than 1 typically represents a distribution that has a larger proportion of both very small and very large values compared with an exponential and is therefore even more “bursty” than an exponential). We also performed the same analysis to amplitude values, computed by taking the square-root of power. The square-root of an exponentially distributed random variable has a Rayleigh distribution, which has a CV of ~0.53. For all the methods, the amplitude CVs were ~0.53 or higher at most frequencies (the plot looked similar to Fig. 2 but was centered at ~0.53; not shown).

To apply the technique used by Feingold and colleagues at different frequencies, we shifted the center frequency of the band-pass filter in steps of 10 Hz. In their method, the band-pass filtered time series was smoothed using a Hann window whose length increased with decreasing center frequency (see materials and methods), thereby increasing the correlation in power values across time and reducing the variability at low frequencies (Fig. 2, green trace). However, at gamma frequencies and above, the smoothing effect was negligible, and the CV was at or above the theoretical value of 1. CV values approached 1 even at lower frequencies if this smoothing operation was not performed, as in the case of HT.

Overall, these results show that, irrespective of the method used, the estimated gamma power is distributed exponentially and therefore trivially appears bursty with occasional large values dispersed between many small values.

#### Matching pursuit-based gamma duration estimation gives accurate results.

We implemented a MP (Mallat and Zhang 1993; Durka et al. 2001; Chandran KS et al. 2016)-based algorithm to compute the duration of the gamma rhythm (see materials and methods for details). To compare the performance of this algorithm with previous techniques, we first generated synthetic LFP by injecting Gabor waveforms of known duration into the spontaneous LFP such that the power spectra of the real and synthetic LFP were matched in the gamma range (see materials and methods). Figure 3*A* shows the power spectral density (PSD) of data obtained from one site in *Monkey 1* during spontaneous activity (magenta trace) and stimulus period (black), along with the PSDs of the simulated LFP with different burst lengths (which varied from 50 ms to 1 s; some of which are shown in different shades of brown). For this electrode, there was a ~14-fold increase in gamma power.

For burst determination, we used a threshold value that was set to a fraction of the mean difference in power between the stimulus and spontaneous periods (by default, the fraction was set to 0.5, yielding threshold of ~7 for this electrode). This threshold value determined the burst centers for all the burst duration algorithms used in this study other than the MP-based algorithm: time points at which the power exceeded this threshold were chosen as seeds and the durations of bursts were computed around those seeds by using the techniques detailed in materials and methods. For the MP algorithm, we selected atoms whose coefficient in the stimulus period exceeded the mean of the largest coefficient in the spontaneous activity in each trial multiplied by the square-root of the threshold fraction (square-root was used because we applied the threshold criterion to amplitude, not power).

Figure 3*B* shows the median of estimated length vs. the injected burst length in the synthetic LFP when a single burst was injected per trial. The CGT-based duration estimators critically depend on the length of the CGT window (compare blue and cyan traces), and plateaued beyond 200 ms. For very short durations, the estimated length increased with injected length, although the slope was much less than unity [consistent with Fig. 4 of Xing et al. (Xing et al. 2012), represented as black rectangles in panel B]. For the algorithm used by Feingold et al. (green trace), the estimated duration increased with injected duration, but the duration was mildly overestimated. The HT-based algorithm (yellow trace) gave duration values similar to those of the algorithm used by Feingold et al. (Feingold et al. 2015). The burst durations estimated using WT-based estimator (purple trace) were close to the actual durations. The MP-based algorithm (red trace) also detected the burst durations robustly. Results were similar when multiple bursts (of the same length) were injected per trial (Fig. 3*C*). Overall, all methods except the CGT-based method gave reasonably accurate results in this case.

To test whether our results depended on the choice of the threshold, we varied the threshold fraction to 0.1 (Fig. 3*D*), 0.25 (Fig. 3*E*), or 0.75 (Fig. 3*F*). Whereas the MP-based algorithm was able to estimate the duration properly at all threshold values (Fig. 3, *D*–*F*), the remaining methods severely underestimated the duration at low thresholds (Fig. 3*D*).

In our data, the visual stimuli were optimized to maximize the gamma power, yielding very large gamma oscillations. To test the performance of different burst detection methods when the rhythm was weaker, we constructed another set of synthetic LFP, in which the injected burst power was 25% of the original, i.e., a ~3.5-fold increase (Fig. 3*G*). In this case, all the burst detectors except the MP-based duration estimator underestimated gamma durations (Fig. 3, *H* and* I*).

As described above, for MP we used the average of the largest coefficient in each trial during the spontaneous period to obtain the threshold. We did not use the average of the coefficients of all the atoms in the spontaneous period, because the total number of atoms depends on the number of iterations used in the MP algorithm. Specifically, when a large number of iterations are used, MP eventually selects atoms of very small magnitudes at all frequency ranges, effectively reducing the average value of the coefficients in each range. Because of this, we used only the largest atom in each trial. However, it is possible that because of this procedure the overall threshold levels were higher in MP, leading to better performance.

To test this, we injected a single burst (per trial) of 400 ms duration and plotted the number of bursts detected by different methods at different threshold fractions (Fig. 4*A*). If MP had used a threshold that was too high, we would have expected that the average number of detected bursts would decrease at higher threshold fractions, but that was not the case. Furthermore, MP correctly detected a single burst over a much wider range of threshold fractions, whereas other methods started picking up a large number of (spurious) bursts at low thresholds. Moreover, a plot of the distribution of burst lengths for different methods (Fig. 4, *B*–*F*) revealed that although the spuriously detected bursts were mainly of short duration for all the other methods, they were more evenly distributed across durations for MP. Since the spurious burst durations were not biased toward low values in the case of MP, even in the presence of this noise the mean durations remained accurate. Results were similar when we injected a burst of 100 ms duration instead of 400 ms (data not shown).

Figure 5 shows the gamma bursts detected using different methods from synthetic signals with short (Fig. 5*A*) and long (Fig. 5*G*) injected gamma bursts (marked with a red ellipse). The time-frequency change in power spectrum using CGT (Fig. 5, *B* and* H*), WT (Fig. 5, *C* and* I*), and MP (Fig. 5, *F* and* L*) and the instantaneous power computed using Feingold’s method (Fig. 5, *D* and* J*) and HT (Fig. 5, *E* and* K*) of the corresponding synthetic signals are also shown, along with the detected gamma lengths (indicated with black, white, and magenta lines; different colors are used only for visual clarity). MP tracked both short and long gamma bursts accurately. When the gamma durations were long, the CGT method estimated the long burst as a group of short bursts, because the phase consistency was not maintained for long durations. For the remaining methods that detected bursts based only on amplitudes (Feingold et al., WT, and HT), the main issue was that, although the true burst was accurately captured, these methods also identified several small spurious bursts as gamma oscillations. We discuss these issues in more detail in the discussion.

#### Gamma durations estimated using MP-based algorithm were larger in real LFP data.

Figure 6 shows gamma bursts identified in a representative trial of stimulus-driven LFP recorded from a single site in *Monkey 1* using all five estimators. All the time-frequency (for CGT, WT, and MP) or power vs. time (for Feingold and HT) plots showed transient increases in power (represented by blobs in the time-frequency spectra) at approximately the same locations in time. However, some of the transients were much sharper in the case of MP, because they were captured by highly transient functions available in the MP dictionary. Also, in the case of MP, many of these transients, despite having sufficient power in the gamma range, did not have center frequencies in the gamma range (often their centers were in the high-gamma range or at zero; see discussion for details) and therefore did not count as gamma bursts. More importantly, MP was able to capture gamma bursts that were not well represented in other methods (e.g., the burst highlighted in white in Fig. 6*F*). To explain this, we first filtered the signal over 40–60 Hz using a fourth-order Butterworth filter to isolate the gamma component of the signal (Fig. 6*G*, blue trace). This signal had larger magnitude at approximately the same locations where power transients were observed in all the methods; however, the phase progression of the signal was consistent across such transients such that a single long gamma atom (Fig. 6*G*, red trace) explained more energy in the signal than two or more short gamma atoms (which were also present in the dictionary and would have been preferred if the phase consistency had not been maintained). Thus, the MP algorithm was able to pick out longer bursts of gamma despite transient changes in the power within the burst duration, whereas other methods necessarily terminated the burst when the power decreased.

We computed gamma durations for 65 sites in *Monkey 1* and 39 sites in *Monkey 2*, with a threshold fraction set to 0.5. Median gamma durations obtained using MP were 332 and 408 ms for *Monkeys 1* and* 2*, respectively, as opposed to 184 and 144 ms using CGT, 208 and 218 ms using the algorithm by Feingold and colleagues, 136 and 174 ms using WT (note that these numbers depend on the standard deviation of the Gaussian used in the Morlet wavelet; see materials and methods for details), and 152 and 120 ms using HT (standard error, computed using bootstrapping, was less than 6 ms for all cases). Median gamma duration computed at a site using MP was not significantly correlated with the change in gamma power at that site (Spearman’s ρ = 0.25, *P* = 0.04 for *Monkey 1*; ρ = 0.22, *P* = 0.18 for *Monkey 2*; significance tested at α = 0.01 after Bonferroni correction for the number of methods; Fig. 7*A*). For the remaining methods, significant correlations were observed in some cases (such as CGT and HT for both monkeys and WT for *Monkey 2*), although the overall magnitude of the burst duration did not vary drastically with power (Fig. 7*A*). The number of detected bursts per trial was ~4 for MP and ~4.5 for Feingold’s methods for both monkeys, whereas with CGT, WT, and HT methods we obtained ~11, ~8.5, and ~6.5 bursts for *Monkey 1* and ~15, ~9.5, and ~8 bursts for *Monkey 2*, respectively. Apart from the CGT-based duration estimator, for which the burst duration rarely exceeded 450 ms, all other methods gave burst durations between 25 and 2,000 ms, but MP yielded a much higher proportion of long bursts (Fig. 7*B*).

The gamma range used here includes the line-noise frequency (50 Hz). Our recordings were done in a heavily shielded Faraday cage that greatly reduced the power line artifact (see Fig. 1*A*; no online or offline notch filter was applied to our data). Nonetheless, to completely rule out any possible influence of line-noise artifacts in our results, we computed the median burst duration at each gamma center frequency separately. Line-noise artifact is typically represented by long atoms in MP, which could have inflated the median gamma duration at and around 50 Hz. However, no inflation was observed (Fig. 7*C*).

Finally, we tested whether gamma duration depended on stimulus contrast. Because the standard error of median was small for all methods (<6 ms), the median gamma length was significantly different in all cases (χ^2^ > 47.5, *P* < 10^−10^ for all methods and both monkeys, Kruskal-Wallis test for comparison of medians, with contrast as the factor). However, a consistent reduction in burst duration with decreasing stimulus contrast in both monkeys was observed only for MP, whereas the other methods showed inconsistent trends across monkeys (Fig. 7*D*). In general, the stimulus contrast did not have a large effect on burst duration: for all methods, even at 25% stimulus contrast, the median gamma duration was >70% of the full contrast condition.

## DISCUSSION

We reviewed existing methods to estimate the duration of a rhythm in the LFP and showed that the “bursty” nature of the rhythm reported in the literature could simply be due to the inherent variability in the spectral estimator, which has no biological significance. We then used a method based on the MP algorithm that overcomes this hurdle and showed that this method is able to robustly estimate the duration of a rhythm in simulated data. Applying this technique to real data, we showed that the duration of the gamma rhythm in the LFP is several hundred milliseconds, much longer than previously reported estimates.

It is interesting to note that longer bouts of gamma oscillations (~250 ms) have been reported in electrocorticogram (ECoG) recordings, in which a large electrode is placed subdurally on the surface of the brain (Rols et al. 2001). However, it is difficult to directly compare gamma durations in LFP and ECoG recordings, since ECoG represents the average activity of a larger cortical area. It is possible that several local oscillators generate short bursts of gamma, but these oscillators are so connected that their bursts are phase aligned, such that the summed activity (captured in the ECoG) shows a more sustained gamma rhythm. Also, in their data, the gamma duration varied considerably between the two monkeys, with their second monkey showing burst durations similar to that reported in LFP recordings (mean duration of 88 ms, as opposed to 254 ms for *Monkey 1*).

### 

#### Performance of previous techniques.

Most of the previous techniques detected bursts based only on the power values estimated using different methods (WT, Feingold’s method, and HT), whereas the CGT-based method detected bursts based on power but then estimated the burst duration based on phase consistency. We found that when gamma power was high, the power-based approaches performed well. This is because when power was high, despite the variability in the estimate of power, the magnitude typically remained above threshold. Furthermore, although the distribution was exponential when computed over long time intervals, power values were correlated over short time scales that depended on the lengths of the windows used in the computation of power. The main issue with the power-based methods is that occasional large power values due to the large variability create spurious seeds, causing a large number of spurious short-duration gamma bursts (Fig. 5). This problem was more severe when the gamma power was low or when a low threshold was used. This issue can be partially addressed by using a more stringent threshold, although in that case it is likely that many valid physiological gamma bursts will also get discarded.

Another way to address this issue is to reduce the variability of single trial power estimates, which could be done, for example, by using a multi-taper (Babadi and Brown 2014; Bokil et al. 2010; Park et al. 1987; Thomson 1982) approach with several tapers, since the variability of the spectral estimate reduces as 1/K, where K is the number of tapers used. For example, Lunqvist et al. (Lundqvist et al. 2016) used the multi-taper method to compute the spectrogram followed by smoothing, which corresponded to effectively using two to three tapers (see their experimental procedures for details). Therefore, their results are less likely to be affected by the issues highlighted here. Similarly, although the estimated power using the technique used by Feingold et al. was highly variable in the gamma frequency range, beta power was found to be much less variable (Fig. 2); therefore, their results are also likely to be less affected by the issues highlighted here.

In contrast, the method used by Xing et al. (2012) relied on consistency in phase, not power across time (although the seed points were chosen based on power and therefore were likely to be affected by the variability in the spectral estimator; see materials and methods for details). We are not aware of a detailed theoretical analysis of the variability in phase estimation with a direct spectral estimator, although we found that while using CGT the delinearized phases did not remain consistent beyond the duration of the CGT window, such that the estimated duration critically depended on the window length and did not substantially increase even when the true rhythm duration increased substantially (Fig. 3). Consequently, a single long burst that was accurately captured using power-based methods was instead estimated as a series of short bursts when using the CGT-based method (Fig. 5*H*).

Using CGT, we obtained gamma durations ~160 ms, as opposed to ~120 ms by Xing et al. There could be several reasons behind this discrepancy. First, they had used drifting gratings whereas we used static gratings, and gamma could potentially be more transient for a drifting grating. However, the difference could just have been due to the calibration procedure adopted by them. Specifically, Xing et al. accounted for the discrepancy between actual and estimated durations by fitting a line between the simulated and estimated durations (their Fig. 4, represented here by black dots in Fig. 3*B*) and “calibrating” the estimated durations in real data using this line (their uncalibrated durations were ~150–175 ms, similar to our durations using their method). We have not followed this calibration procedure, because the linear relationship was not maintained once the simulated length increased beyond 200 ms (Fig. 3).

#### Advantages of the MP-based duration estimator.

To the best of our knowledge, this study is the first to use MP to detect gamma bursts in LFP data. MP algorithms have been previously used in EEG recordings to separate transients from oscillations (Jmail et al. 2011) and to detect epileptiform activity-related transients (Goelz et al. 2000; Jmail et al. 2011), high-frequency oscillations (Chaibi et al. 2013), or slow waves (Picot et al. 2011). Many of these studies also compared the MP-based method with other techniques (especially WT), although the issue with the variability in the spectral estimator was not highlighted.

There are several reasons why MP outperformed other methods discussed here. First, the issue with the variability in spectral estimation is largely avoided because, unlike other methods, power is not computed at consecutive time points. Note that, although the MP algorithm appears to bypass the issue with the variability in spectral estimator by performing all computations in the time domain, the computation of the inner product as described in *Eq. 5* is actually very similar to spectral estimation. For example, in short-time Fourier transform (STFT), the spectral estimate at a particular frequency (that yields the amplitude and phase) is precisely the inner product of the signal with a complex sinusoid multiplied by an appropriate window (see Chandran KS et al. 2016 for a comparison between MP and STFT using a common “filter-bank” framework). The key difference is that in previous methods spectral estimation is done at multiple time points (to compute the power and phase), and the burst length is determined based on the consistency of the estimates across time. In contrast, in MP, many different functions of different durations are available at any given time point, and the function with the best duration (which has the largest inner product with the residue) is chosen for representation. Since we did not rely on the consistency of spectral estimate across time (as done in other methods), we were able to get a more robust estimate of the rhythm duration.

Second, the MP-based estimate uses fewer parameters than other methods. Note that all methods (including MP) need a threshold value to identify the center of a burst. However, for WT, Feingold’s method, and HT, this threshold also directly influenced the burst length, because the length determined was based on when the power went below half the threshold. For the method used by Xing et al., the burst duration was estimated based on phase deviation, but an arbitrary cutoff of 45° was used, and changing this value would have changed the estimated duration. For MP, however, once an atom is selected, its duration does not depend on any other parameter and is therefore more resilient toward variations in the choice of a threshold (Figs. 3 and 4).

Third, MP is able to better resolve transients into functions that do not have center frequencies in the gamma band. As shown in Fig. 6, the LFP has a lot of transients that have power at all frequencies, including the gamma band. If this power is above the threshold, spurious bursts are obtained. In MP, such sharp transients are often captured with functions that have a center frequency of zero (in which case the function is simply a Gaussian) or at frequencies beyond the gamma range (see Ray et al. 2008; Ray 2015) for separating transients from oscillations based on this property). Thus, although such transients have power in the gamma band, they do not always contribute toward gamma bursts.

Fourth, whereas most of the spurious bursts have very small durations for all the other methods, for MP the durations are more evenly distributed (Fig. 4*F*); therefore, the mean burst durations are not as biased toward smaller values.

Finally, the MP-based algorithm is more tolerant toward transient fluctuations in the amplitude or phase of the rhythm. The CGT-based approach imposes the strictest condition on the rhythm, essentially testing whether gamma acts like a “clock” or a metronome, since it explicitly checks for phase consistency. Amplitude-based methods (WT, Feingold's, and HT) impose a weaker condition, where inconsistent phase progression can be tolerated as long as the power remains high. MP allows further relaxation, whereby minor fluctuations in power/phase can be tolerated (as shown in Fig. 6). For example, if the rhythm has a long duration but has a “gap” in between (because of a transient reduction in amplitude or change in phase), the overall signal structure can still be best explained by a single long Gabor function rather than two short Gabors. In other methods, the burst is instead necessarily terminated when the amplitude or phase changes abruptly. Whether a rhythm with transient “breaks” in between can still provide a reliable reference frame depends on the proposed computational framework and specific details of how a rhythm modulates spiking activity; a metronome precision may not be necessary for some of the proposed functions (Fries et al. 2007; Nikolić et al. 2013).

#### Limitations of the MP-based duration estimator.

Although our MP based method outperformed previous methods, there are some limitations. First, our simulations yielded good results for MP partly because the injected gamma bursts were Gabor atoms (i.e., had a Gaussian taper) that matched the shape of the functions available in the MP dictionary. If there were a mismatch, for example, if the gamma oscillations started or ended abruptly, our MP based method could miscalculate the duration (for example, in our data the estimated gamma duration occasionally exceeded the stimulus duration, which could have been because the real gamma stopped more abruptly than the tapering assumed by a Gaussian function). However, Gabor gamma bursts are likely to be well suited for all the other methods as well. Both CGT and WT (using Morlet wavelet) use Gabor bases that match the shape of the burst. For the filter-based methods (Feingold’s and HT), the raw signal is first filtered by a smooth Hann function before computing the power. Power of a signal that has an abrupt onset or offset cannot be captured appropriately using these techniques, since the convolution with the filter smooths out the transient. Indeed, failure of other methods to accurately estimate the duration of even a smoothly rising and falling gamma burst highlights the strong effect of the variability in the spectral estimator. Gamma bursts with sudden onset or offset cannot be properly estimated using any of the methods discussed here. In the case of MP, however, this issue can be partly addressed by extending the dictionary to include functions that can change more abruptly (for example, sinusoids multiplied by rect or bell-cosine functions of different durations).

Second, since MP is a greedy algorithm that tries to maximize the energy at each iteration, if it selects an “inappropriate” function (which does not represent a biological phenomenon), subsequent iterations yield functions that try to correct this mistake and therefore yield more inappropriate functions (for a concrete example and careful discussion, see Chen et al. 2001).

Third, the MP algorithm is very slow compared with other techniques used in this study. MP is known to have a large convergence time and therefore needs a large number of iterations (Pati et al. 1993). We had to use a stochastic dictionary (Durka et al. 2001) instead of a dyadic dictionary as originally proposed by Mallat and Zhang (1993), so that the durations were not limited to powers of 2, but the implementation with a stochastic dictionary is much slower than with a dyadic dictionary (Ray et al. 2003). In addition, to obtain accurate results, we had to densely pack the time-frequency space with suitable functions (see materials and methods for details). Consequently, our MP algorithm was ~6,600 times slower than that of Xing and colleagues and ~30,000 times slower than that of Feingold and colleagues. One improvement of the MP is the basis pursuit (BP) algorithm (Chen and Donoho 1994; Chen et al. 2001), which decomposes the signal to a given set of waveforms and then iteratively improves the decomposition. Because BP performs a global optimization, it is much faster than MP and could potentially address some of the other concerns as well.

Finally, the Gabor dictionary is appropriate only when the center frequency of the rhythm does not vary with time, which is typically the case for gamma rhythm, for which the center frequency is slightly high at the onset but quickly settles down to a steady value (Fig. 1*A*). In cases where the center frequency varies with time, for example in response to a long odor pulse in the moth olfactory system (Ito et al. 2009), or in response to a grating whose contrast varies with time (Ray and Maunsell 2010), filtering-based methods (Feingold’s and HT) or WT may work better than MP employing Gabor bases.

#### Behavioral significance of gamma bursts.

Similar to the study of Xing et al. (2012) who recorded from anesthetized animals, our study also did not involve any manipulation of behavior, since our animals were passively fixating. Since gamma has been associated with a variety of cognitive functions such as attention (Fries et al. 2001), an important question is whether the duration of gamma rhythm could be dependent on the attentional load. While that question is beyond the scope of this study, we note that the effect of attention on neuronal responses as well as gamma rhythm is typically small in V1; previous studies have shown that gamma center frequency increases by 2–3 Hz and is accompanied by a slight reduction in power (Bosman et al. 2012; Chalk et al. 2010). Therefore, it is unlikely that the duration of gamma would change drastically with a change in the focus of attention.

Although the duration of the gamma rhythm in our data set is longer than what has been reported in previous studies, we do not make any claim about its functional significance, which would need a more detailed understanding about the computational timescales over which neurons process information and how rhythms could influence this processing. For visual processing, computational time scales could be comparable to the duration between two microsaccades, which is ~300 ms in primates (Bosman et al. 2009). A recent study reported consistent signs of gamma phase across sites in V1 computed over 300-ms windows and showed that the local phase of gamma exerted a spatially asymmetric directed effect on the firing rate of a nearby population, such that the phase variations of gamma could potentially change the direction of communication in V1 (Besserve et al. 2015). On the other hand, for working memory tasks, even brief bursts of gamma (<100 ms) could be sufficient (Lundqvist et al. 2016).

In our data set, although the median gamma duration was >300 ms, the mode gamma duration was shorter (Fig. 7*B*), suggesting that most of the gamma bursts were short but there were occasional bouts of long gamma bursts. Moreover, median gamma durations were long only when the analysis was done after stimulus transient. If the analysis was restricted to 0–500 ms, the median gamma burst durations obtained using MP were shorter (~83 and ~179 ms for the two monkeys), which was expected because the stimulus onset-related sharp transients in the signal had energy in gamma range as well (see Fig. 1*A*) and were therefore captured using atoms of short durations. Thus, while we have shown that gamma rhythms can be sustained in some cases, whether such long bouts of gamma could play a role in cognitive processing is not addressed here and is beyond the scope of this study. However, whether or not gamma plays an active role in cognition, analysis of its evolution in time could provide important clues about the properties of the underlying network.

## GRANTS

Wellcome Trust DBT India Alliance (500145/Z/09/Z; Intermediate Fellowship to S. Ray). Tata Trusts Grant (to S. Ray). DBT-IISc Partnership Program (to S. Ray). Department of Science and Technology – Intensive Research in High Priority Areas (to C. S. Seelamantula).

## DISCLOSURES

No conflicts of interest, financial or otherwise, are declared by the authors.

## AUTHOR CONTRIBUTIONS

S.C.K., C.S.S., and S.R. conceived and designed research; S.C.K. performed experiments; S.C.K. and S.R. analyzed data; S.C.K., C.S.S., and S.R. interpreted results of experiments; S.C.K. and S.R. prepared figures; S.C.K. and S.R. drafted manuscript; S.C.K., C.S.S., and S.R. edited and revised manuscript; S.C.K., C.S.S., and S.R. approved final version of manuscript.
